# *Brucella suis *urease encoded by *ure*1 but not *ure*2 is necessary for intestinal infection of BALB/c mice

**DOI:** 10.1186/1471-2180-7-57

**Published:** 2007-06-19

**Authors:** Aloka B Bandara, Andrea Contreras, Araceli Contreras-Rodriguez, Ana M Martins, Victor Dobrean, Sherry Poff-Reichow, Parthiban Rajasekaran, Nammalwar Sriranganathan, Gerhardt G Schurig, Stephen M Boyle

**Affiliations:** 1 Department of Biomedical Sciences & Pathobiology, Virginia Polytechnic Institute and State University, Blacksburg, Virginia 24061, USA

## Abstract

**Background:**

In prokaryotes, the ureases are multi-subunit, nickel-containing enzymes that catalyze the hydrolysis of urea to carbon dioxide and ammonia. The *Brucella *genomes contain two urease operons designated as *ure*1 and *ure*2. We investigated the role of the two *Brucella suis *urease operons on the infection, intracellular persistence, growth, and resistance to low-pH killing.

**Results:**

The deduced amino acid sequence of urease-α subunits of operons-1 and -2 exhibited substantial identity with the structural ureases of alpha- and beta-proteobacteria, Gram-positive and Gram-negative bacteria, fungi, and higher plants. Four *ure *deficient strains were generated by deleting one or more of the genes encoding urease subunits of *B. suis *strain 1330 by allelic exchange: strain 1330Δ*ure*1K (generated by deleting *ureD *and *ureA *in *ure*1 operon), strain 1330Δ*ure*2K (*ureB *and *ureC *in *ure*2 operon), strain 1330Δ*ure*2C (*ureA*, *ureB*, and *ureC *in *ure*2 operon), and strain 1330Δ*ure*1KΔ*ure*2C (*ureD *and *ureA *in *ure*1 operon and *ureA*, *ureB*, and *ureC *in *ure*2 operon). When grown in urease test broth, strains 1330, 1330Δ*ure*2K and 1330Δ*ure*2C displayed maximal urease enzyme activity within 24 hours, whereas, strains 1330Δ*ure*1K and 1330Δ*ure*1KΔ*ure*2C exhibited zero urease activity even 96 h after inoculation. Strains 1330Δ*ure*1K and 1330Δ*ure*1KΔ*ure*2C exhibited slower growth rates in tryptic soy broth relative to the wild type strain 1330. When the BALB/c mice were infected intraperitoneally with the strains, six weeks after inoculation, the splenic recovery of the *ure *deficient strains did not differ from the wild type. In contrast, when the mice were inoculated by gavage, one week after inoculation, strain 1330Δ*ure*1KΔ*ure*2C was cleared from livers and spleens while the wild type strain 1330 was still present. All *B. suis *strains were killed when they were incubated *in-vitro *at pH 2.0. When the strains were incubated at pH 2.0 supplemented with 10 mM urea, strain 1330Δ*ure*1K was completely killed, strain 1330Δ*ure*2C was partially killed, but strains 1330 and 1330Δ*ure*2K were not killed.

**Conclusion:**

These findings suggest that the *ure*1 operon is necessary for optimal growth in culture, urease activity, resistance against low-pH killing, and *in vivo *persistence of *B. suis *when inoculated by gavage. The *ure*2 operon apparently enhances the resistance to low-pH killing *in-vitro*.

## Background

A number of environmentally and medically important bacteria produce the enzyme urease (urea amidohydrolase) [1], which catalyzes the hydrolysis of urea, leading to the production of carbamate and ammonia. In an aqueous environment, the carbamate rapidly and spontaneously decomposes to yield a second molecule of ammonia and one of carbonic acid. The carbonic acid equilibrates in water, as do the two molecules of ammonia, which become protonated to yield ammonium hydroxide ions. The reaction results in an increased pH of the environment [reviewed in [2-5]]. In sites where microorganisms colonize epithelial surfaces, such as the normal flora of the oral cavity or intestines, or when certain pathogenic bacteria infect tissues, the metabolism of urea by microbial ureases can have a profound impact on tissue integrity, microbial ecology, and the overall health of the host.

The ureases of most microbes are composed of three subunits α, β, and γ that are encoded by *ureA*, *ureB *and *ureC *genes respectively. The plant jack bean produces a single-subunit urease [12], whereas, in gastroduodenal pathogen *H. pylori*, the *ureA *and *ureB *genes are sufficient to encode urease. Nevertheless the UreAB subunits of *H. pylori *can be aligned with the UreABC subunits of other ureolytic bacteria and with the single polypeptide of the jack bean urease. The crystal structure of the *Klebsiella aerogenes *urease reveals a trimeric configuration [13]. Biochemical analyses of ureases by gel filtration have shown that other bacterial ureases are multimeric and probably have similar stoichiometry [4].

Ureases are structurally complex enzymes, and additional urease subunits are required for the production of a catalytically active holoenzyme *in-vivo*. Ureases are among the few enzymes that require nickel for activity. Biogenesis of a functional urease in prokaryotes requires the presence and expression of four urease accessory genes, *ureDEFG*. *In vitro *experiments using purified accessory proteins support the idea that UreE likely acts as a carrier of nickel [14] and that UreDFG form a chaperone-like complex that keeps the apoenzyme in a configuration competent to accept nickel [15].

Urease activity can be a critical factor in the colonization, persistence and pathogenesis of bacteria. Considering the products produced by urease, it would be logical to assume that one of the enzyme's functions is to allow nitrogen assimilation. In fact, urea represents an assimilable nitrogen source for bacteria that can colonize the human body and there is evidence suggesting that ammonia assimilation from urea occurs *in-vivo*. A significant proportion of the urea produced in the liver ends up in the intestines, where it can be hydrolyzed and assimilated by several different species of anaerobic, ureolytic bacteria [3]. Similarly, the oral bacterium, *Actinomyces naeslundii *can use urea as a primary nitrogen source for growth [6]. So there is little doubt that nitrogen acquisition as the result of urease activity can be important in the ecology of complex populations colonizing the human body. However, it is an open question as to whether the capacity to assimilate ammonia produced by urease contributes to the pathogenic potential of bacteria. Instead, it appears that the release of the strongly alkaline ammonia released by urease is a major cause of the damage to the host tissue, and in some cases, a key factor in persistence of pathogens [reviewed in [2]]. Jubier-Maurin *et al.*, (30) identified the *nikABCDE *operon encoding the specific transport system for nickel in *B. suis*. Insertional inactivation of *nikA *strongly reduced the activity of the nickel metalloenzyme urease, which was restored by addition of nickel excess. Intracellular growth rates of the *B. suis *wild-type and *nikA *mutant strains in human monocytes were similar, indicating that *nikA *was not essential for this type of infection.

The Brucellae are gram-negative, facultative intracellular bacterial pathogens of a wide range of vertebrates [7]. This pathogen is the etiologic agent of the disease brucellosis and the pathological manifestations of brucellosis include abortion and sterility in animals [7], and meningitis, endocarditis, spondylitis and arthritis in humans [8]. Paulsen *et al*., [9] annotated the genome of *B. suis *strain 1330 (biovar 1), and discovered that unlike many other organisms, *Brucella *have two urease gene operons located on chromosome I (GenBank accession no. NC_004310). Urease activity is important for the nitrogen assimilation and persistence of other bacterial species like *Helicobacter pylori *[10,11]. We investigated the role of the two *B. suis *urease operons on the infection, intracellular persistence, growth, and resistance to low-pH killing. We report that the *B. suis ure*1 operon, in contrast to *ure*2, appears to be principally responsible for determining urease activity, optimum growth and resistance to low-pH killing *in-vitro *and persistence *in-vivo*.

## Results

### Organization, and nucleotide and amino acid sequences of urease genes

**Figure 1:**
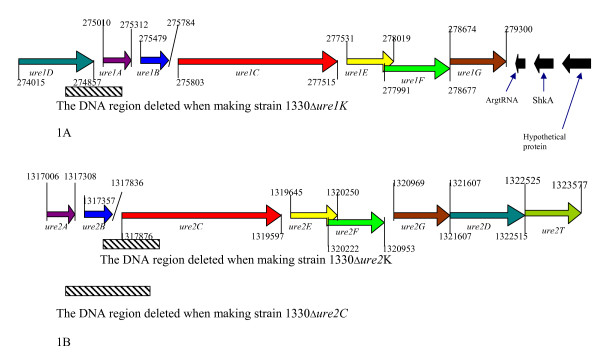
The schematic representations of the *ure *operons with corresponding Ure subunits, and deletion sites of mutant strains 1330Δ*ure*1K, 1330Δ*ure*2K and 1330Δ*ure*2C. A: *ure*1 operon. B: *ure*2 operon. The numbers represent the location of the genes in the chromosome I.

The *ure*1 and *ure*2 operons are located on the chromosome I of *B. suis *strain 1330 (GenBank accession number NC_004310). The *ure*1 operon is 5284-bp long and composed of seven coding sequences (CDS). The *ure*2 operon is 6571-bp long and comprised eight CDS (Figure 1). The *ureA *gene was the same in size in both operons (302-bp). All the other genes of *ure*2 operon were slightly longer than their counterparts in *ure*1 operon. The *ureC *gene was the longest in each operon (1712-bp in operon-1 and 1721-bp in operon-2). The G+C content of each *ure *gene was compared with that of its counterpart of the other operon and found not differ substantially between *ure *genes of operon-1 and operon-2 (Table 1). The identity of each *ure *gene was compared with that of its counterpart of the other operon. The u*reA*, *ureB*, *ureC*, and *ureG *genes of the two operons exhibited 52 to 60% identity, whereas the *ureD*, *ureE*, and *ureF *genes did not share significant identity (Table 1).

**Table 1:** Sequence identities between the two *B. suis *urease operons

G+C Content			Gene comparison	Identity (%)
		
Gene	Operon-1	Operon-2		
*ureA*	60.3	57.3	*ure1A *vs *ure2A*	52
*ureB*	58.4	58.1	*ure1B *vs *ure2B*	60
*ureC*	60.4	59.3	*ure1C *vs *ure2C*	55
*ureD*	62.0	58.1	*ure1D *vs *ure2D*	23
*ureE*	59.3	58.9	*ure1E *vs *ure2E*	Not significant
*ureF*	63.3	59.1	*ure1F *vs *ure2F*	Not significant
*ureG*	57.4	56.5	*ure1G vs ure2G*	54
*ureT*	-	60.4	-	-

The deduced amino acid sequences encoded by the *ureA*, *ureB*, and *ureC *genes in both operons displayed great identity with the structural urease subunits of a vast range of organisms including Gram-positive bacteria, Gram-negative bacteria, photosynthetic bacteria, fungi, and higher plants (see Table 5). For instance, urease subunits of other organisms exhibited up to 81% identity with the UreC of *ure*1 and up to 69% identity with the UreC of *ure*2. The ureases of alpha and beta-proteobacteria exhibited the greatest identity with UreC of operon-1, whereas, the ureases of all species of *Yersinia *exhibited the greatest identity with the UreC of operon-2.

Real-time PCR assays produced amplicons in sizes exactly similar to the expected sizes for each *ure *gene (data not shown). The UreB subunit in the *ure*2 operon contains a predicted hydrophobic signal sequence and suggests that the subunit may localize in the periplasmic space. All other Ure subunits lack any signal sequences and were predicted to localize in the cytoplasm (data not shown).

### Genomic characterization of generated mutant *B. suis *strains

Four mutant *B. suis *strains were generated by allelic exchange, i.e., 1330Δ*ure*1K, 1330Δ*ure*2K, 1330Δ*ure*2C, and 1330Δ*ure*1KΔ*ure*2C. The PCR assays produced a predicted 2.2-kb amplicon from the wild type *B. suis *strain 1330 and an approximately 3.2-kb amplicon from the mutant strain 1330Δ*ure*1Kwith the ureONE-Forward and ureONE-Reverse primers (see Table 6); a predicted 2.2-kb-size amplicon from the strain 1330 and an approximately 2.8-kb product from strain1330Δ*ure*2K with ureTWO-Forward and ureTWo-Reverse primers; and a predicted 2.9-kb-sizeamplicon from the strain 1330 and an approximately 3.4-kb product from strain1330Δ*ure*2C with primers Ure-2-AB-Forward and Ure-2-AB-Reverse. The PCR assays with the primer pairs ureONE-Forward/ureONE-Reverse and Ure-2-AB-Forward/Ure-2-AB-Reverse confirmed that the double-mutant strain 1330Δ*ure*1KΔ*ure*2C carried a 575-bp deletion from the *ure1DA *region and a 1.2-kb deletion from the *ure2ABC *region (Figures 1A and 1B).

### Expression of urease, urease enzyme activity and growth rates of *B. suis *strains

**Figure 2:**
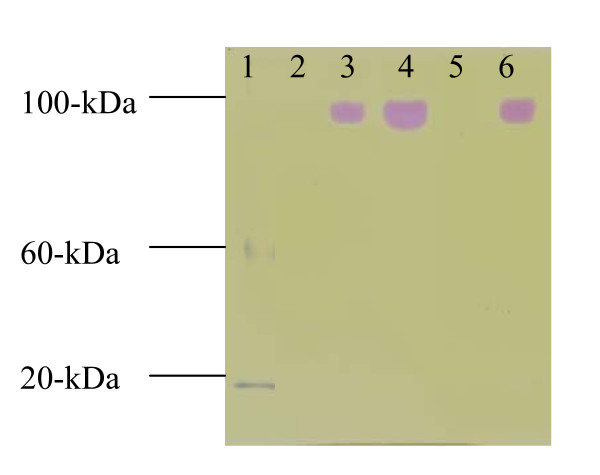
Native 8% polyacrylamide gel assay with *B. suis *extracts. Lanes-1: ladder; 2: *B. suis *strain 1330Δ*ure*1K; 3: strain 1330Δ*ure*2K; 4: strain 1330Δ*ure*2C; 5: strain 1330Δ*ure*1KΔ*ure*2C; and 6: strain 1330.

Native polyacrylamide gel electrophoresis revealed urease activity at approximately 95-kDa from strains 1330, 1330Δ*ure*2K and 1330Δ*ure*2C, but not from strains 1330Δ*ure*1K or 1330Δ*ure*1KΔ*ure*2C (Figure 2).

**Figure 3:**
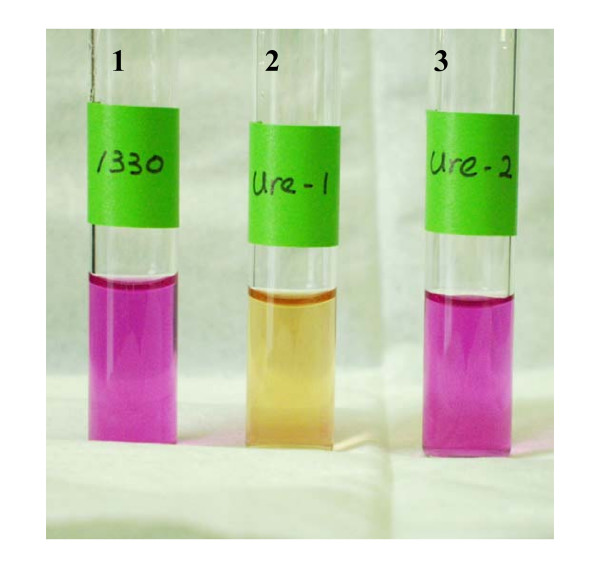
Urease test broth 24 hours after inoculation with *B. suis *strains. Tube-1: strain 1330 (positive), 2: 1330Δ*ure*1K (negative), and 3: 1330Δ*ure2*K (positive).

In a quantitative urease assay, mutants 1330Δ*ure*1K and 1330Δ*ure*1KΔ*ure*2C exhibited 0 activity, the wild type and the mutant 1330Δ*ure*2K displayed maximal activity, and mutant 1330Δ*ure*2C showed slightly reduced activity (Table 2). In qualitative urease assay, urease test broth started turning positive within 4 h after either strain 1330, 1330Δ*ure*2K, or 1330Δ*ure*2C were introduced, and acquired a bright pink color after approximately 24 h (Figure 3 and Table 2). In contrast, strains 1330Δ*ure*1K and 1330Δ*ure*1KΔ*ure*2C failed to cause a pink color in the urease test broth even after 96 h of incubation (Figure 3 and Table 2).

**Table 2:** *B. suis *strains: generation time (doubling time, h) in TSB and urease activity in urease test broth.

Strain	Doubling time (h)*	Urease activity	
		
		Qualitative**	Quantitative***
1330	5.1	+	9.28
1330Δ*ure*1K	5.8	-	~0
1330Δ*ure*2K	5.3	+	9.38
1330Δ*ure*2C	5.1	+	8.28
1330Δ*ure*1K1330Δ*ure*2C	6.5	-	~0

*A representative experiment was used to calculate the generation time.

** + represents positive or pink color and – represents negative or yellow color of the uninoculated test broth; the color remained unchanged even 96 h after inoculation

***Specific activity μmoles/min/mg of protein.

Strains 1330Δ*ure*1K and 1330Δ*ure*1KΔ*ure*2C, both urease negative, grew approximately 25% slower than wild type strain 1330. In contrast, strains 1330Δ*ure*2K and 1330Δ*ure*2C, both urease positive, did not display any measurable differences in growth rate compared to strain 1330 (Table 2).

### Survival of *B. suis *strains in macrophage cell lines

When used to infect J774A.1 or H36.12a [Pixie 12a] mouse macrophage cell lines, the recovery of all the *B. suis *strains declined 2–3 log_10 _cfu between 0 and 4 h post-inoculation. During the next 20 h, all the *B. suis *strains increased 1–2 log_10 _cfu. There were no significant differences between the wild type and the urease mutant strains in terms of their ability to replicate in macrophages (data not shown).

### Survival of *B. suis *strains in BALB/c mice

**Figure 4:**
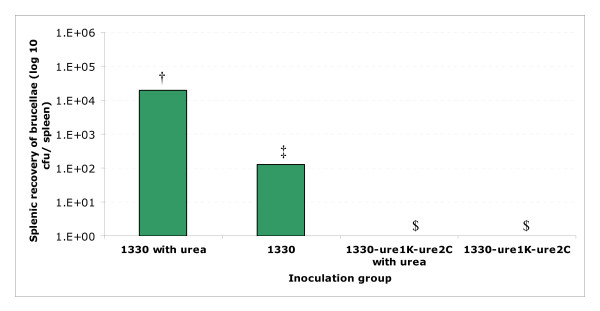
Recovery of *Brucella *cfu from spleens one week after BALB/c mice were inoculated by gavage with wild type strain 1330 or strain 1330Δ*ure*1KΔ*ure*2C with or without urea supplementation. *P *value for the difference among mean values was <0.01. The mean values that share the same symbol do not differ from one another significantly; and the mean values designated by different symbols differ from one another significantly.

**Figure 5:**
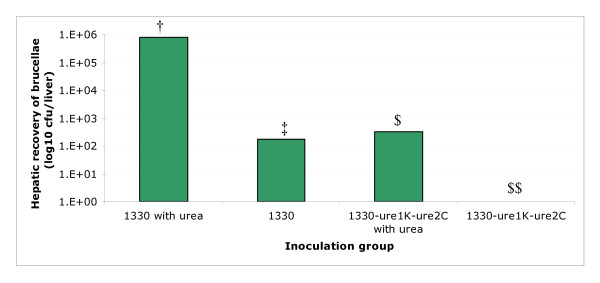
Recovery of *Brucella *cfu from livers one week after BALB/c mice were inoculated by gavage with the wild type strain 1330 or strain 1330Δ*ure*1KΔ*ure*2C with or without urea supplementation. *P *value for the difference among mean values was <0.025. The mean values that share the same symbol do not differ one another significantly; and the mean values designated by different symbols differ one another significantly.

Following an intraperitoneal inoculation, the recovery of *ure *mutants from spleens did not differ from the wild type strain at 6 wks post-infection (Table 3). When the mice were inoculated by gavage, one week after inoculation, strain 1330 was recovered from spleens (Figure 4) as well as from livers (Figure 5). When the mice were inoculated with strain 1330 supplemented with 10 mM urea, nearly 2.2 log_10 _greater cfu was recovered from spleens and nearly 3.5 log_10 _greater cfu was recovered from livers. However, when the mice were inoculated with strain 1330Δ*ure*1KΔ*ure*2C, with or without urea supplementation, no cfu were recovered from spleens (Figure 4) but nearly 2.5 log_10 _cfu was recovered from livers only when the inoculum was supplemented with 10 mM urea (Figure 5).

**Table 3:** Splenic recovery of *B. suis *strains six weeks after intraperitoneal inoculation in BALB/c mice

Strain	Injected dosage (log_10 _cfu/mouse)	cfu 6 weeks after inoculation (log_10_/spleen)^a^
Trial-1		
1330 (wild)	5.24	4.41 ± 0.18†
1330Δ*ure*1K	5.24	4.61 ± 0.20†
1330Δ*ure*2K	5.22	4.40 ± 0.18†
1330Δ*ctpA *(control)	5.25	2.04 ± 0.89‡
		
Trial-2		
1330 (wild)	4.11	4.25 ± 0.23
1330Δ*ure*2C	4.16	4.17 ± 0.25
1330Δ*ure*1KΔ*ure*2C	4.28	4.19 ± 0.31

^a^In Trial-1:*P *value for the difference among mean values was <0.005. The mean value of a strain designated by a dagger (†) is significantly different from the mean value of a strain designated by a double dagger (‡) but is not significantly different between strains designated by the same symbol.

In Trial-2: The observed significance level among mean values was < 0.1 <*P *< 0.9.

### Resistance of *B. suis *strains against low-pH killing

**Figure 6:**
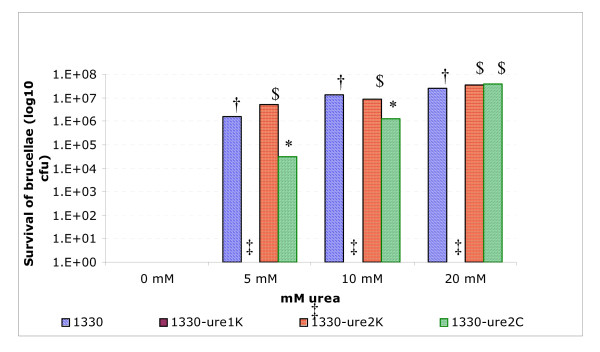
Survival of *B. suis *strains 1330, 1330Δ*ure*1K, 1330Δ*ure*2K, and 1330Δ*ure*2C after incubation at pH 2.0 with or without urea. At each urea concentration, the *P *value for the difference among mean cfu was <0.005. At each urea concentration, the mean values designated by different symbols differ one from another significantly; and the mean values that share the same symbol do not differ one from another significantly.

The wild type and the *ure *mutants did not differ with respect to the survival after 90 min incubation at pH 4.0 or 7.0 (data not shown). All the strains including the wild type were killed when incubated at pH 2.0 for 90 min (Figure 6). When the strains were supplemented with 5 mM urea during incubation at pH 2.0, more than 6.0 log_10 _cfu of strains 1330 and 1330Δ*ure*2K were recovered. In comparison to strain 1330, the recovery of the strain 1330Δ*ure*2C was nearly 1.5 log_10 _lower at 5 mM urea concentration and nearly 1.0 log_10 _lower at 10 mM urea. In contrast to strains 1330, 1330Δ*ure*2K and 1330Δ*ure*2C, strain 1330Δ*ure*1K was not recovered after incubation at pH 2.0 supplemented at any urea concentration (Figure 6). Addition of urea did not change the pH of the incubation media.

## Discussion

The *ure*A, *ure*B, and *ure*C genes of *B. suis *(Figure 1) encode the γ, β, and α subunits respectively, and the urease holoenzyme of *B. suis *is likely to be assembled in a trimeric configuration. The total predicted mass of the *B. suis *urease holoenzyme (UreA+B+C) is 91-kDa. The native polyacrylamide gel reveals urease activity at approximately 95-kDa (Figure 2) and supports a trimeric configuration of this enzyme. The *Brucella *genome also contains *ure*DEFG genes (Figure 1) in each of *ure*1 and *ure*2 operons and are predicted to produce the UreD, UreE, UreF and UreG proteins. Unlike many other microorganisms, *Brucella *contains two operons encoding urease subunits (Figure 1) located on the chromosome I. Based on the similarities of G+C contents among genes in *ure1 *and *ure2 *operons, it is unlikely that any of the operons were acquired by horizontal gene transfer. The genes of the *ure*1 operon shared less than 60% identity with their counterparts of the *ure*2 operon (Table 1). In particular, the *ureE *and *ureF *genes of the *ure*1 operon did not share considerable similarity with those genes in the *ure*2 operon. Based on the relatively low identity among genes between *ure*1 and *ure*2 operons, it seems unlikely that they were the result of a recent duplication event. However, further analyses are required to confirm these predictions.

We generated a series of mutants by disrupting the first few genes encoding structural subunits of each urease. All seven genes of *ure1 *operon appear to be transcribed in a single direction. The gaps between individual *ure *genes are extremely small (Figure 1), so that all or most of the genes are possibly expressed under a single, common promoter – leading to a polycistronic mRNA. The *ure*G is the last gene of the *ure1 *operon. All three genes downstream of the *ure*G are transcribed in the opposite direction, from the complementary strand (Figure 1). The closest non-*ure *gene to *ureG *encodes an Arg-tRNA. The genes downstream of this tRNA gene encode a serine histidine kinase (CDS *Shk*) and a hypothetical protein. The distance from the stop codon of *ureG *to the stop codon of Arg-tRNA (transcribed in opposite direction) is 124-bp. Thus it does not seem likely that the insertion of the antibiotic resistance gene into *ure1 *influences the expression of genes downstream of the operon. In addition, it is not apparent that these genes have any regulatory role on urease expression.

The deletion of genes within the *ure*1 operon caused the disappearance of urease activity on a native polyacrylamide gel (Figure 2). Even though the *ure2 *operon was present in the *ure1 *mutant, it failed to produce urease activity as measured by either of two assays. In addition, a disruption of the *ure2 *operon did not have any impact on production of a detectable urease activity.

The deletion of genes within the *ure*1 operon caused the slower growth and loss of urease activity (Table 2) suggesting that the genes are necessary for maximal growth and urease activity of *B. suis*. Strains 1330Δ*ure*2K and 1330Δ*ure*2C made by deleting genes of *ure*2 operon did not display any change in growth rate or urease activity, suggesting that the genes of *ure*2 operon are not required for these functions. Overall, *B. suis *is apparently capable of exhibiting urease activity even without involvement of *ure*2 operon. This observation raises the question as to whether the genes within *ure*2 are actually being expressed. Our measurements using RT-PCR of RNA extracted from *B. suis *1330 cultured in TSB (data not shown) suggest that all the genes in *ure*2 are being expressed. It is apparent that much more work will have to be done to determine the role of the *ure*2 operon. Except for a detectable role in resistance to acidic pH *in-vitro*, there were no other detectable phenotypes associated with the *ure*2 mutants under the various conditions employed. One obvious question is whether there is any post-translational interaction going on between the subunits of both *ure *operons. Given that there is no urease activity in an *ure*1 mutant, it is possible to conclude that the corresponding Ure2 subunits are not acting to restore Ure1 activity.

In addition to the ureolytic bacteria [3] that can use urea as a primary nitrogen source for growth, the urinary tract pathogen *Ureaplasma ureolyticum *[16] and some alkalophilic bacteria [17] can use ureolysis to generate ATP. Urea, particularly at millimolar concentrations can readily enter the cell [4]. Thus, in bacteria that utilize urea for nitrogen assimilation or ATP generation, the urease activity is expected to occur intracellularly. In most organisms, the ureases are found in the cytoplasm, although there is a report of urease membrane association and cell surface localization as well [4]. The ureases for which primary sequence information is available do not have characteristics consistent with being integral membrane proteins or secreted through the general secretory pathways. Consistent with the ureases of other bacteria, the deduced *B. suis ure *encoded subunits (with exception of UreB encoded by the *ure*2 operon) are predicted to localize in the cell cytoplasm (data not shown). Thus it may be possible that urease functions within the cytoplasm in order to assimilate nitrogen. Further work is needed to characterize the exact location of urease subunits and whether they play a role in nitrogen uptake.

The *ure *mutants did not exhibit any decline in persistence in BALB/c mice when inoculated intraperitoneally (Table 3), suggesting that the urease activity is not critical once the pathogen has entered the host. Using *ure *mutants of *Bordetella bronchiseptica*, Monack and Falkow [18] reported that urease is not essential for the colonization of the guinea-pig respiratory and digestive tracts. One of the common ways that humans acquire brucellosis is through consumption of contaminated milk. *Brucella *needs to have a mechanism to resist the low-pH killing in the gastrointestinal tract. When the mice were inoculated by gavage, the wild type strain was recovered from livers and spleens, whereas, strain 1330Δ*ure*1KΔ*ure*2C was not. It is noteworthy that when the infecting doses of *B. suis *were supplemented with urea, the recovery of strain 1330 from livers and spleens was increased, but the mutant strain 1330Δ*ure*1KΔ*ure*2C was recovered only from livers. These findings suggest that the urease activity and sufficient substrate is needed for *B. suis *to cope with the low pH in the gastrointestinal tract i.e. either stomach or upper intestine. Similar observations have been recently reported by Sangari et al., [37] in *ure*-mutant strains of *B. abortus*. Thus, similar to the urease of *H. pylori *[10,11], that of *B. suis *appears to be a factor in coping with the pH of the gastrointestinal tract. Even though the *ure1, ure2 *mutant *B. suis *exhibited no urease activity, urea supplementation raised the recovery of this strain from livers. This is possible because other bacterial species in stomach and gastro-intestinal tract may have utilized the supplemented urea to reduce the acidity and facilitated enhanced survival of *Brucella*. It is worth noting that inoculation via an oral route versus direct inoculation in the stomach may be more representative of a natural infection. However, we chose to deliver a known dose of *Brucella *and measure uptake through the gastro-intestinal tract (i.e., into the spleen and liver).

For an intracellular bacterium like *Brucella *that replicates inside phagocytic cells associated with the various tissues including the reproductive tract [19], urease may not be important for regulating the pH in an intracellular milieu. Correspondingly, the lack of differences between wild type and *ure *mutants inoculated intraperitoneally during splenic clearance was not surprising (Table 3). Nevertheless, in the case of *H. pylori *and perhaps other organisms, urease is an important factor in survival in severely acidified environments, probably because neutralization of the extracellular milieu around the cells is needed to prevent irreversible membrane damage. Acidification and subsequent alkalinization in the phagosomal compartment is a prerequisite for a successful *Brucella *infection *in-vitro *[20]. However, the amount of urea present in macrophages maybe low enough as not to cause a significant pH change. As such, the impact of urease activity on macrophage pH can be considered insignificant, and therefore, similar survival rates in macrophages between wild-type and *ure *mutants was not unexpected.

## Conclusion

The *B. suis *genome contains two operons encoding urease. The genes in the *ure*1 operon are important for optimal growth in culture, and indispensable for urease activity, resistance to low-pH killing and survival of the pathogen when inoculated by gavage. The genes in the *ure*2 operon seem to be dispensable for the above functions, but slightly enhance the resistance to low-pH killing measured *in-vitro*. The apparent lack of urease activity encoded by *ure2 *is consistent with the observation by Hooper and Berg who reviewed over 20 microbial genomes with respect to gene innovation following gene duplication (38). They concluded that most gene copies are deleted but of the ones retained, they function in weak or ancillary roles. Thus it is possible that *ure*2 in *B. suis *is diverged enough to encode a new function that has yet to be defined.

## Methods

### DNA and protein sequence analyses

The nucleotide sequence of the urease genes was analyzed with DNASTAR software (DNASTAR, Inc., Madison, Wis.). The destination of the deduced proteins upon translation and processing was predicted using the Subloc v1.0 server of the Institute of Bioinformatics of the Tsinghua University . Identity of the *ureA*, *ureB*, and *ureC *genes of *B. suis ure1 *and *ure2 *operons with sequences of the EMBL/GenBank/DDBJ databases was analyzed using the BLAST software [22] at the National Center for Biotechnology Information (Bethesda, MD).

### Bacterial strains, plasmids, and reagents

*B. suis *strain 1330 was obtained from our culture collection. *Escherichia coli *strain Top10 (Invitrogen Life Technologies, Carlsbad, Calif.) was used for producing plasmid constructs. *E. coli *were grown in Luria-Bertani (LB) broth or on LB agar (Difco Laboratories, Sparks, MD). *Brucella *were grown either in Trypticase soy broth(TSB) or on Trypticase soy agar (TSA) plates (Difco) at 37°C in the presence of 5% CO_2 _as previously described [23]. Theplasmids used in this study are listed in Table 4. Bacteria containing plasmids were grown in the presence of ampicillinor kanamycin at a 100-μg/ml concentration (Table 4).

**Table 4:** Description of the plasmids and bacterial strains used in this study

Plasmid or strain	Description	Source or reference
Plasmids		
pCR2.1	TA cloning vector, 3.9-kb, Amp^r^	Invitrogen
pCR*ure*1	pCR2.1 with 2.2-kb insert containing the *B. suis ure*1D, *ure*1A, *ure*1B, *ure*1C genes; Amp^r^	This study
PCR*ure2*ABC	pCR2.1 with 2.2-kb insert containing the *B. suis ure*2A, *ure*2B, *ure*2C genes; Amp^r^	This study
pGEM-3Z	Cloning vector, 2.74-kb, Amp^r^	Promega
pGEM*ure*1	pGEM-3Z with 2.2-kb insert containing the *B. suis ure*1D, *ure*1A, *ure*1B, and *ure*1C genes; Amp^r^	This study
pGEM*ure2*ABC	pGEM-3Z with 2.2-kb insert containing the *B. suis ure*2A, *ure*2B, and *ure*2C genes; Amp^r^	This study
pGEM*ure2*ABC-2	pGEM-3Z with 2.9-kb insert containing the *B. suis ure*2A, *ure*2B, *ure*2C genes; Amp^r^	This study
pUC4K	Cloning vector, 3.9-kb, Kan^r^, Amp^r^	Pharmacia
pGEM*ure1*K	pGEM*ure*1 with 0.9-kb *Nco*I fragment deleted and blunt ended and a 1.6-kb *Pvu*II-cut and blunt-ended Kan^r ^cassette from pUC4K ligated, Kan^r^, Amp^r^	This study
pGEM*ure2*ABCK	pGEM*ure2 *with 0.6-kb *Sac*II fragment deleted and blunt ended and a 1.6-kb *Pvu*II-cut and blunt-ended Kan^r ^cassette from pUC4K ligated, Kan^r^, Amp^r^	This study
pGEM*ure2*ABCC	pGEM*ure2 *with 1.2-kb *Cla*I and *Mfe*I fragment deleted and blunt ended and a 1.7-kb *Eco52*I plus *Kpn*I-cut and blunt-ended Cm^r ^cassette from pBBR4MCS ligated, Cm^r^, Amp^r^	This study
pBBR4MCS	Broad-host-range vector; Cm^r^	[27]
Strains		
*Escherichia coli *Top10		
	F^- ^*mcrA Δ*(*mrr-hsd*RMS-*mcr*BC) Φ80*lac*ZΔM*15 Δlac*X74 *deo*R*rec*A1 *ara*D139 Δ(*ara-leu*) *7697 gal*U *gal*K *rps*L (StrR) *end*A1 *nup*G	Invitrogen
*Brucella suis*		
1330	Parent-type, smooth strain	G.G. Schurig
1330Δ*ure1*K	*ure1*DA deleted mutant of 1330, Kan^r^	This study
1330Δ*ure2*K	*ure2*BC deleted mutant of 1330, Kan^r^	This study
1330Δ*ure2*C	*ure2*ABC deleted mutant of 1330, Cm^r^	This study
1330Δ*ure1*KΔ*ure2C*	*ure1*DA and *ure2*ABC deleted mutant of 1330, Cm ^r^, Kan^r^	This study

All experiments with live *Brucella *were performed in a Biosafety Level 3 facility in the Infectious Disease Unit of the Virginia-Maryland Regional College of Veterinary Medicine per standard operating procedures approved by the Centers for Disease Control and Prevention.

### Recombinant DNA methods

Genomic DNA was isolated from *B. suis *strain 1330 by use ofa QIAGEN blood and tissue DNA kit (QIAGEN Inc., Valencia, CA). Plasmid DNA was isolated using either plasmid Mini- or Midiprep purification kits (QIAGEN). Restriction digests, Klenow reactions, and ligations of DNA were performed as described elsewhere [24]. Restriction enzymes, Klenow fragment, and T4 DNA ligase enzyme were purchased from Promega Corporation (Madison, WI). Ligated plasmid DNAwas transformed into *E. coli *Top10 cells by heat shock per the guidelines of the supplier (Invitrogen). Plasmid DNA was electroporated into *B. suis *with a BTX ECM-600electroporator (BTX, San Diego, CA), as described previously[25].

### Mutation of *ure*1 operon

A 2,241-bp region including the whole length of the *ure1D*, *ure1A*, and *ure1B *genes and a portion of *ure1C *gene (Figure 1) was amplified via PCR using the genomic DNA of *B. suis *strain 1330 and the primers UreaseONE-Forward and UreaseONE-Reverse (RansomHill Bioscience, Inc., Ramona, CA) (see Table 6). The amplified gene fragment was cloned into the pCR2.1 vector of the TA cloning system (Invitrogen) to produce plasmid pCR*ure1*. From this plasmid the *ure1 *region was isolated by *EcoR*I digestion and cloned into pGEM-3Z (Promega) and the resulting 5.0-kb plasmid was designated pGEM*ure1*. The suicide vector pGEM*ure1*K was constructed as follows: the plasmid pGEM*ure1 *was digested with *Nco*I to delete a 575-bp region from the *ure1 *region. The *Nco*I sites on the 4.4-kb plasmid were filled in by reaction with Klenow enzyme and ligated to the1.6-kb *Pvu*II fragment of pUC4K (also blunt ended) containing the Tn*903 npt *gene [26], which confers kanamycin resistance(Kan^r^) to *B. suis*. The resulting suicide vector was designated pGEM*ure1*K. The *E. coli *Top10 cells carrying the recombinant plasmid were picked from TSA plates containing kanamycin (100 μg/ml).

One ug of pGEM*ure1*K was used to electroporate *B. suis *strain 1330; several colonies of strain 1330 were obtained from a TSA plate containing kanamycin (100 μg/ml). These colonies were streaked on TSA plates containing ampicillin (100 μg/ml) to determine whether a single- or double-crossover event had occurred. Five of the colonies did not grow on ampicillin-containingplates, suggesting that a double-crossover event had occurred. PCR with the primers UreaseONE-Forward and UreaseONE-Reverse (see Table 6) confirmed that a double-crossover event had taken place in all five transformants. One of these strains was chosen for further analyses and designated 1330Δ*ure*1K.

### Mutation of *ure2B *and *ure2C*

A 2,214-bp region including the whole length of the *ure2A *and *ure2B *genes, and a portion of the *ure2C *gene was amplified via PCR using the primers UreaseTWO-Forward and ureaseTWO-Reverse (see Table 6). The amplified gene fragment was cloned into the pCR2.1 vector to produce plasmid pCR*ure2ABC*. From this plasmid, the *ure2*ABC region was isolated by *BamH*I and *Xba*I digestion and cloned into the same sites of plasmid pGEM-3Z (Promega). The resulting 5.0-kb plasmid was designated pGEM*ure2*ABC. The suicide vector pGEM*ure2*ABCK was constructed as follows: the plasmid pGEM*ure2*ABC was digested with *Sac*II to delete a 940-bp region from the *ure2*ABC region, sticky sites filled in with Klenow enzyme, and ligated to the 1.6-kb *Pvu*II fragment of pUC4K. The resulting suicide vector was designated pGEM*ure2*ABCK. One microgram of pGEM*ure2*ABCK was used to electroporate *B. suis *strain 1330, and a transformed strain containing a double-crossover event was verified by PCR and designated 1330Δ*ure*2K.

### Mutation of *ure2A*, *ure2B *and *ure2C*

A 2,923-bp region including the whole length of *ure2*A and *ure2*B genes, and a portion of *ure2*C gene (Figure 1) was amplified via PCR using the primers Ure-2-AB-Forward and Ure-2-AB-reverse (see Table 6). The amplified gene fragment was purified using a Qiagen PCR purification kit (Qiagen), digested with *BamH*I and *Xba*I, cloned into the same sites of plasmid pGEM-3Z (Promega) to produce the 5.7-kb plasmid pGEM*ure2*ABC-2. The suicide vector pGEM*ure2*ABCC was constructed as follows: the plasmid pGEM*ure2*ABC-2 was digested with *Cla*I and *Mfe*I to delete a 1215-bp region from the *ure2*ABC region, and the ends were filled in with Klenow enzyme. The 1.7-kb gene encoding resistance to chloramphenicol (Cm^r^) was isolated by digesting the plasmid pBBR1MCS [27] with *Eco52*I plus *Kpn*I, and the ends were filled in with Klenow enzyme. The larger fragment of the plasmid pGEM*ure2*ABC-2 was ligated with the Cm^r ^gene, to make the suicide vector pGEM*ure2*ABCC. One ug of pGEM*ure2*ABCC was used to electroporate *B. suis *strain 1330 and the transformants were picked from TSA plates containing chloramphenicol (30 μg/ml). A transformed *B. suis *strain with a double-crossover event was verified by PCR and designated 1330Δ*ure*2C.

### Generation of an *ure1*, *ure2 *strain

One ug of suicide vector pGEM*ure2*ABCC was used to electroporate mutant *B. suis *strain 1330Δ*ure*1K. The transformants were picked from TSA plates containing kanamycin (100 μg/ml) plus chloramphenicol (30 μg/ml). A transformant *B. suis *strain with a double-crossover event was verified by PCR and designated 1330Δ*ure*1KΔ*ure*2C.

### Real-time PCR assays

RNA was isolated from broth cultures of *B. suis *strain 1330 by the procedure described previously [31]. After a 75% ethanol wash, the dried RNA pellet was resuspended in RNase- and DNase-free water (Sigma). The concentration of the RNA was be determined with the RiboGreen RNA Quantitation kit (Molecular Probes). Genomic DNA was digested with RNase-free DNase (Ambion), and precipitated with GlycoBlue (Ambion). RNA samples not treated with reverse transcriptase was also subjected to PCR to measure the level of contamination from genomic DNA. For each RNA sample, the control transcript (*sig*A) and the target mRNA were reverse-transcribed using the ReverTra Dash kit (Toyobo). The cDNA was amplified using the Light Cycler (Roche) in conjunction with the DNA Master SYBR Green I kit (Roche). Primers specific for the internal standard of *ure *genes were purchased from Sigma-Genosys. The target cDNA was normalized internally to the *sigA *cDNA level in the same sample [32,33].

### Growth rates of *B. suis*

Single colonies of strains 1330, 1330Δ*ure*1K, 1330Δ*ure*2K, 1330Δ*ure*2C and 1330Δ*ure*1KΔ*ure*2C were grown at 37°C for 72 h to stationary phase in 5 ml of TSB. These cultures were used to inoculate 25 ml of TSB in a Klett side-arm flask to 12 to 16 Klett units. Cultures were grown at 37°C at 200 rpm; Klett readings were recorded every 2 h in a Klett-Summerson colorimeter (New York, NY).

### Native polyacrylamide gel electrophoresis

Extracts were prepared from *B. suis *strains 1330,1330Δ*ure*1K, 1330Δ*ure*2K, 1330Δ*ure*2C and 1330Δ*ure*1KΔ*ure*2C using glass beads and vortex, in Tris-HCl 30 mM, pH 8.0). Buffer without SDS or mercaptoethanol were added to the extracts and the extracts were loaded into the gel that did not contain SDS. After running was complete, the gel was placed in 0.02% cresol red-0.1 EDTA, washed several times until it became yellow, and incubated with 1.5% urea at room temperature until pink bands appeared, i.e., the positive urease reaction.

### Urease enzyme activity of strains-qualitative

Fifty μl  of culture (grown for 72 h in TSB) volumes of strains 1330, 1330Δ*ure*1K, 1330Δ*ure*2K, 1330Δ*ure*2C and 1330Δ*ure*1KΔ*ure*2C were used to inoculate 5 ml volumes of urease test broth (Difco). The contents were incubated at 37°C with 200 rpm shaking. At 8, 24 and 48 hours after incubation, the cultures were centrifuged to remove the cells. The color change in the clarified urease test broth was measured using a Klett-Summerson colorimeter. The native color of the urease test broth was used as a blank.

### Urease enzyme activity of strains-quantitative

The Coomassie Brilliant Blue G, TRIS, NADPH, 2-oxoglutarate and glutamate dehydrogenase (from bovine liver) were from Sigma-Aldrich. Urea was obtained from Qiagen. All other reagents were of analytical grade.

The concentration of protein in the extracts was determined using a Bradford-modified assay [34]. All assays (final volume of 2 mL) were performed in 31 mM Tris-HCl pH 8.0 buffer at 28°C, using a Beckman DU 800 UV/Vis spectrophotometer, with a stirred, temperature-controlled multi-cell holder. Urease activity was determined using a coupled assay with glutamate dehydrogenase [35] Glutamate dehydrogenase 12 U/mL was incubated with 250 μM NADPH for 5 min. 2-Oxoglutarate (1 mM) and *B. suis *extracts were added and the reaction was followed at 340 nm. The observed decrease in absorbance monitored during this period is due to nonspecific oxidation of NADPH by several enzymes in the extract. When the absorbance was stable, urea (10 mM) was added and the decrease in absorbance at 340 due to urease activity was measured for 5 min. Initial rates were calculated from the linear portion of the curves, by linear regression using the least squares method. The absorption coefficient used for NADPH was 6.22 M^-1^cm^-1 ^[36]. The volume of extracts was varied and the specific activity of urease was calculated. One unit of urease activity was defined as the amount of enzyme that hydrolyzes 1 μmol of urea per min. Specific activities were calculated as units of urease per mg of protein in the extract.

### Preparation of *B. suis *infection innocula

TSA plates were inoculated with single colonies of *B. suis *strains. After 4 days of incubation at 37°C with 5% CO_2_, the cells were harvested from plates, washed with phosphate-buffered saline(PBS), resuspended in 20% glycerol, and frozen at -80°C. The number of viable cells or cfu was determined by counting after spreading of dilutions of the cell suspensions on TSA that were incubated at 37C with 5% CO_2_. The cultures from these were used to inoculate mice and macrophages as described below.

### Persistence of recombinant *B. suis *strains in macrophages

The mouse macrophage-like cell lines J774 and H36.12a [Pixie 12a] were obtained from the American Type Culture Collection (Manassas, VA). The macrophage cells were seeded at a density of 5 × 10^5^/ml in Dulbecco's modified essential medium (DMEM) (Sigma-Aldrich) into 24-well tissue culture dishes and cultured at 37°C with 5% CO_2 _until confluent. The tissue culture medium was removed, 200 μl (10^8 ^cells) of the bacterial suspension in PBS was added, and the cells were incubated at 37°C for 4 h. The suspension above the cell monolayer was removed, and the macrophages washed three times with PBS. One milliliter of DMEM containing 25 μg of gentamicin was added, and the cells were incubated for 48 h at 37°C. At various time points (0, 1, 4, 24, and 48 h of incubation), the growth medium was removed, the cells were washed with PBS, and 500 μl of 0.25% sodium deoxycholate was added to lyse the infected macrophages. After 5 min the lysate was diluted in PBS, and the number of *B. suis *cfu was determined after growth at 37°C with 5% CO_2 _for 72 h on TSA. Triplicate samples were taken at all time points, and the assay was repeated two times.

### Survival of *B. suis *strains inoculated intraperitoneally

The Animal Care Committee of the Virginia Polytechnic Institute and State University approved the procedures used in handling research animals. Six-week-old female BALB/c mice (Charles River Laboratories, Wilmington, MA) were allowed 1 week of acclimatization. Groups of seven or eight mice each were intraperitoneally injected with 4.1 to 5.2 log_10 _cfu of *B. suis *strains 1330,1330Δ*ure*1K, 1330Δ*ure*2K, 1330Δ*ure*2C and 1330Δ*ure*1KΔ*ure*2C. Six weeks after inoculation, mice were killed using CO_2 _asphixiation, and the *Brucella *cfu per spleen determined as described previously [23]. Briefly, spleens were collected and homogenized in TSB. Serial dilutions of each splenic homogenate were plated on TSA and the number ofcfu was determined after 4 days of incubation at 37°C with 5% CO_2_.

### Survival of *B. suis *strains in spleens and liver when inoculated by gavage

Groups of seven to eight BALB/c mice each were dosed by gavage with 8.6 log_10 _cfu of *B. suis *strain 1330 or strain 1330Δ*ure*1KΔ*ure*2C, with or without supplementation of 10 mM urea in approximately 0.5 ml PBS. Mice were sacrificed 1 week after inoculation, and the *Brucella *cfu per spleen or per liver was determined as described previously [23].

### *In-vitro *pH sensitivity of strains

The pH of the phosphate buffered saline (PBS) was adjusted to 2, 4 or 7 by adding 1N HCl. The urea concentration of the PBS was adjusted to 5, 10, or 20 mM by adding urea. The PBS with different pH and urea contents were inoculated with 8.0 log_10 _cfu of strains 1330,1330Δ*ure*1K, 1330Δ*ure*2K or 1330Δ*ure*2C, and incubated at 37°C. At the end of 90 min incubation, serial dilutions of each culture were plated on TSA. The number of cfu on plates was determined after 4 days of incubation at 37°C with 5% CO_2_.

### Data analyses

The mean and the standard deviation values from the mouse tissue clearance studies were calculated using the Microsoft Excel 2001 program (Microsoft Corporation). The Student *t *test was performed in the analysis of cfu data in the macrophage study. The cfu data from the splenic and liver clearance studies, and *in vitro *pH sensitivity study were analyzed by performing analysis of variance. The mean cfu counts among treatments were compared using the least-significance pair-wise comparison procedure [29].

## Authors' contributions

AB and VD constructed mutant strains, and carried out part of growth and splenic clearance assays. AC carried out part of splenic and hepatic clearance assays and acid tolerance assays. SP and PR carried out macrophage assays. GS, NS and SB conceived of the study and participated in its design and coordination. AC-R carried out native gel electrophoresis, and AM performed quantitative urease activity.

**Table 5:** Identity of B. suis urease-1α and urease-2α sequences with the urease α subunits or  urease proteins in GenBank

Accession number With urease-1α	Organism	Identity%
YP_221060.1|	*Brucella abortus*	99%
NP_540569.1|	*Brucella melitensis*	99%
NP_105696.1|	*Mesorhizobium loti*	81%
AAL83830.1|	*Rhizobium leguminosarum*	78%
NP_386576.1|	*Sinorhizobium meliloti*	79%
NP_355353.1|	*Agrobacterium tumefaciens*	78%
YP_166953.1|	*Silicibacter pomeroyi*	75%
EAQ05022.1|	*Oceanicola batsensis*	75%
BAB21067.1|	*Rhodobacter capsulatus*	75%
ZP_01056362.1|	*Roseobacter sp.*	73%
ZP_00962028.1|	*Sulfitobacter sp.*	73%
YP_109255.1|	*Burkholderia pseudomallei*	70%
AAA25151.1|	*Klebsiella Aerogenes*	68%
ZP_00990659.1|	*Vibrio splendidus*	69%
NP_286680.1|	*Escherichia coli*	67%
NP_886007.1|	*Bordetella parapertussis*	66%
ZP_00504504.1|	*Clostridium thermocellum*	63%
NP_176922.1|	*Arabidopsis thaliana*	64%
YP_248248.1|	*Haemophilus influenzae*	62%
NP_391545.1|	*Bacillus subtilis*	62%
ZP_00133792.2|	*Actinobacillus Pleuropneumoniae*	62%
ABC74584.1|	*Yersinia enterocolitica*	57%
AAK32714.1|	*Helicobacter pylori*	59%
NP_336355.1|	*Mycobacterium tuberculosis*	62%
AAZ99164.1|	*Streptococcus vestibularis*	57%
AAO85883.1|	*Glycine max*	63%
BAB78715.1|	Oryza sativa	62%
XP_750204.1|	*Aspergillus fumigatus*	62%
		
With urease-2α		
NP_539564.1|	*Brucella melitensis*	99%
YP_222047.1|	*Brucella abortus*	99%
ZP_00828648.1|	*Yersinia frederiksenii*	69%
ZP_00797116.1|	*Yersinia pestis *(and all species of *Yersinia*)	69%
NP_929433.1|	*Photorhabdus luminescens*	67%
NP_929433.1|	*Photorhabdus luminescens*	67%
NP_241120.1|	*Bacillus halodurans*	59%
YP_237504.1|	*Pseudomonas syringae*	59%
NP_391545.1|	*Bacillus subtilis*	57%
YP_368247.1|	*Burkholderia sp.*	56%
ZP_00612021.1|	*Mesorhizobium sp.*	57%
NP_533073.1|	*Agrobacterium tumefaciens*	57%
YP_295218.1|	*Ralstonia eutropha*	56%
EAQ70376.1|	*Synechococcus sp.*	57%
105696.1|	*Mesorhizobium loti*	56%
YP_248248.1|	*Haemophilus influenzae*	56%
NP_979959.1|	*Bacillus cereus*	56%
NP_440403.1|	*Synechocystis sp.*	56%
AAG52306.1|	*Arabidopsis thaliana*	57%
ZP_00133792.2|	*Actinobacillus Pleuropneumoniae*	55%
ZP_00990659.1|	*Vibrio splendidus*	56%
NP_286680.1|	*Escherichia coli*	56%
YP_204056.1|	*Vibrio fischeri*	55%
AAP51176.1|	*Helicobacter pylori*	55%
BAB78715.1|	*Oryza sativa*	56%
AAR21273.1|	*Streptococcus thermophilus*	54%
NP_336355.1|	*Mycobacterium tuberculosis*	55%
AAC46128.1|	*Bordetella bronchiseptica*	52%
XP_658035.1|	*Aspergillus nidulans*	54%

**Table 6:** Sequences of primers (5’ to 3’) used to amplify ure genes

Primer name	Sequence (5' to 3')
UreaseONE-Forward	CGACGCCGTAGGTAAATC
UreaseONE-Reverse	TGAAATGGACATGGGTATCG
	
UreaseTWO-Forward	GCTTGCCCTTGAATTCCTTTGTGG
UreaseTWO-Reverse	ATCTGCGAATTTGCCGGACTCTAT
	
Ure-2-AB-Forward	CGGGGATCCCATCACAATCGGCAAACA
Ure-2-AB-reverse	CGGTCTAGAATGGCGCGAAGGAAGGTT 3'

## Supplementary Material

Additional File 1 Identity of *B. suis *urease-1α and urease-2α sequences with the urease α subunits or urease proteins in GenBank. Click here for file

Additional File 2 Sequences of primers (5' to 3') used to amplify ure genes. Click here for file
