# Supplementary material for: Brucella suis urease encoded by ure1 but not ure2 is necessary for intestinal infection of BALB/c mice
Source: BMC Microbiol. 2007 Jun 19;7:57. doi: 10.1186/1471-2180-7-57 (PMC1983905; doi:10.1186/1471-2180-7-57)
Supplement: Additional File 1 — Identity of B. suis urease-1α and urease-2α sequences with the urease α subunits or urease proteins in GenBank. [file 1471-2180-7-57-S1.doc]

**Supplementary Material**

# Table-1. Identity of *B. suis* urease-1α and urease-2α sequences with the urease α subunits or urease proteins in GenBank

#

# Accession number Organism Identity%

# With urease-1α

# [YP_221060.1|](http://www.ncbi.nlm.nih.gov/entrez/query.fcgi?cmd=Retrieve&db=Protein&list_uids=62289267&dopt=GenPept) *Brucella abortus* 99%

[NP_540569.1|](http://www.ncbi.nlm.nih.gov/entrez/query.fcgi?cmd=Retrieve&db=Protein&list_uids=17987935&dopt=GenPept) *Brucella melitensis* 99%

NP_105696.1| *Mesorhizobium loti* 81%

[AAL83830.1|](http://www.ncbi.nlm.nih.gov/entrez/query.fcgi?cmd=Retrieve&db=Protein&list_uids=19070377&dopt=GenPept) *Rhizobium leguminosarum* 78%

NP_386576.1| *Sinorhizobium meliloti* 79%

NP_355353.1| *Agrobacterium tumefaciens* 78%

YP_166953.1| *Silicibacter pomeroyi*  75%

[EAQ05022.1|](http://www.ncbi.nlm.nih.gov/entrez/query.fcgi?cmd=Retrieve&db=Protein&list_uids=84392811&dopt=GenPept) *Oceanicola batsensis* 75%

[BAB21067.1|](http://www.ncbi.nlm.nih.gov/entrez/query.fcgi?cmd=Retrieve&db=Protein&list_uids=12313641&dopt=GenPept) *Rhodobacter capsulatus* 75%

[ZP_01056362.1|](http://www.ncbi.nlm.nih.gov/entrez/query.fcgi?cmd=Retrieve&db=Protein&list_uids=86137786&dopt=GenPept) *Roseobacter sp.* 73%

[ZP_00962028.1|](http://www.ncbi.nlm.nih.gov/entrez/query.fcgi?cmd=Retrieve&db=Protein&list_uids=83953306&dopt=GenPept) *Sulfitobacter sp.* 73%

[YP_109255.1|](http://www.ncbi.nlm.nih.gov/entrez/query.fcgi?cmd=Retrieve&db=Protein&list_uids=53720269&dopt=GenPept) *Burkholderia pseudomallei* 70%

[AAA25151.1|](http://www.ncbi.nlm.nih.gov/entrez/query.fcgi?cmd=Retrieve&db=Protein&list_uids=149338&dopt=GenPept) *Klebsiella Aerogenes* 68%

[ZP_00990659.1|](http://www.ncbi.nlm.nih.gov/entrez/query.fcgi?cmd=Retrieve&db=Protein&list_uids=84387642&dopt=GenPept) *Vibrio splendidus* 69%

NP_286680.1| *Escherichia coli* 67%

[NP_886007.1|](http://www.ncbi.nlm.nih.gov/entrez/query.fcgi?cmd=Retrieve&db=Protein&list_uids=33598364&dopt=GenPept) *Bordetella parapertussis*  66%

[ZP_00504504.1|](http://www.ncbi.nlm.nih.gov/entrez/query.fcgi?cmd=Retrieve&db=Protein&list_uids=67874953&dopt=GenPept) *Clostridium thermocellum* 63%

NP_176922.1| *Arabidopsis thaliana* 64%

[YP_248248.1|](http://www.ncbi.nlm.nih.gov/entrez/query.fcgi?cmd=Retrieve&db=Protein&list_uids=68249136&dopt=GenPept) *Haemophilus influenzae* 62%

NP_391545.1| *Bacillus subtilis* 62%

[ZP_00133792.2|](http://www.ncbi.nlm.nih.gov/entrez/query.fcgi?cmd=Retrieve&db=Protein&list_uids=53729261&dopt=GenPept) *Actinobacillus Pleuropneumoniae* 62%

[ABC74584.1|](http://www.ncbi.nlm.nih.gov/entrez/query.fcgi?cmd=Retrieve&db=Protein&list_uids=85700461&dopt=GenPept) *Yersinia enterocolitica* 57%

[AAK32714.1|](http://www.ncbi.nlm.nih.gov/entrez/query.fcgi?cmd=Retrieve&db=Protein&list_uids=13605430&dopt=GenPept) *Helicobacter pylori* 59%

NP_336355.1| *Mycobacterium tuberculosis*  62%

[AAZ99164.1|](http://www.ncbi.nlm.nih.gov/entrez/query.fcgi?cmd=Retrieve&db=Protein&list_uids=74099907&dopt=GenPept) *Streptococcus vestibularis* 57%

[AAO85883.1|](http://www.ncbi.nlm.nih.gov/entrez/query.fcgi?cmd=Retrieve&db=Protein&list_uids=32170831&dopt=GenPept) *Glycine max* 63%

[BAB78715.1|](http://www.ncbi.nlm.nih.gov/entrez/query.fcgi?cmd=Retrieve&db=Protein&list_uids=17402589&dopt=GenPept) Oryza sativa 62%

XP_750204.1| *Aspergillus fumigatus* 62%

With urease-2α

NP_539564.1| *Brucella melitensis* 99%

YP_222047.1| *Brucella abortus* 99%

[ZP_00828648.1|](http://www.ncbi.nlm.nih.gov/entrez/query.fcgi?cmd=Retrieve&db=Protein&list_uids=77973096&dopt=GenPept) *Yersinia frederiksenii* 69%

[ZP_00797116.1|](http://www.ncbi.nlm.nih.gov/entrez/query.fcgi?cmd=Retrieve&db=Protein&list_uids=77635026&dopt=GenPept) *Yersinia pestis* (and all species of *Yersinia*) 69%

[NP_929433.1|](http://www.ncbi.nlm.nih.gov/entrez/query.fcgi?cmd=Retrieve&db=Protein&list_uids=37526089&dopt=GenPept) *Photorhabdus luminescens* 67%

NP_929433.1| *Photorhabdus luminescens* 67%

NP_241120.1| *Bacillus halodurans* 59%

YP_237504.1| *Pseudomonas syringae* 59%

NP_391545.1| *Bacillus subtilis*  57%

YP_368247.1| *Burkholderia sp.* 56%

[ZP_00612021.1|](http://www.ncbi.nlm.nih.gov/entrez/query.fcgi?cmd=Retrieve&db=Protein&list_uids=69276427&dopt=GenPept) *Mesorhizobium sp.* 57%

[NP_533073.1|](http://www.ncbi.nlm.nih.gov/entrez/query.fcgi?cmd=Retrieve&db=Protein&list_uids=17936283&dopt=GenPept) *Agrobacterium tumefaciens* 57%

[YP_295218.1|](http://www.ncbi.nlm.nih.gov/entrez/query.fcgi?cmd=Retrieve&db=Protein&list_uids=73540698&dopt=GenPept) *Ralstonia eutropha* 56%

[EAQ70376.1|](http://www.ncbi.nlm.nih.gov/entrez/query.fcgi?cmd=Retrieve&db=Protein&list_uids=86169120&dopt=GenPept) *Synechococcus sp.* 57%

[105696.1|](http://www.ncbi.nlm.nih.gov/entrez/query.fcgi?cmd=Retrieve&db=Protein&list_uids=13474128&dopt=GenPept) *Mesorhizobium loti* 56%

[YP_248248.1|](http://www.ncbi.nlm.nih.gov/entrez/query.fcgi?cmd=Retrieve&db=Protein&list_uids=68249136&dopt=GenPept) *Haemophilus influenzae* 56%

[NP_979959.1|](http://www.ncbi.nlm.nih.gov/entrez/query.fcgi?cmd=Retrieve&db=Protein&list_uids=42782712&dopt=GenPept) *Bacillus cereus* 56%

[NP_440403.1|](http://www.ncbi.nlm.nih.gov/entrez/query.fcgi?cmd=Retrieve&db=Protein&list_uids=16329675&dopt=GenPept) *Synechocystis sp.* 56%

[AAG52306.1|](http://www.ncbi.nlm.nih.gov/entrez/query.fcgi?cmd=Retrieve&db=Protein&list_uids=12324683&dopt=GenPept) *Arabidopsis thaliana*  57%

[ZP_00133792.2|](http://www.ncbi.nlm.nih.gov/entrez/query.fcgi?cmd=Retrieve&db=Protein&list_uids=53729261&dopt=GenPept) *Actinobacillus Pleuropneumoniae*  55%

[ZP_00990659.1|](http://www.ncbi.nlm.nih.gov/entrez/query.fcgi?cmd=Retrieve&db=Protein&list_uids=84387642&dopt=GenPept) *Vibrio splendidus* 56%

[NP_286680.1|](http://www.ncbi.nlm.nih.gov/entrez/query.fcgi?cmd=Retrieve&db=Protein&list_uids=15800666&dopt=GenPept) *Escherichia coli* 56%

[YP_204056.1|](http://www.ncbi.nlm.nih.gov/entrez/query.fcgi?cmd=Retrieve&db=Protein&list_uids=59711280&dopt=GenPept) *Vibrio fischeri* 55%

[AAP51176.1|](http://www.ncbi.nlm.nih.gov/entrez/query.fcgi?cmd=Retrieve&db=Protein&list_uids=31580721&dopt=GenPept) *Helicobacter pylori* 55%

[BAB78715.1|](http://www.ncbi.nlm.nih.gov/entrez/query.fcgi?cmd=Retrieve&db=Protein&list_uids=17402589&dopt=GenPept) *Oryza sativa* 56%

[AAR21273.1|](http://www.ncbi.nlm.nih.gov/entrez/query.fcgi?cmd=Retrieve&db=Protein&list_uids=38489172&dopt=GenPept) *Streptococcus thermophilus* 54%

[NP_336355.1|](http://www.ncbi.nlm.nih.gov/entrez/query.fcgi?cmd=Retrieve&db=Protein&list_uids=15841318&dopt=GenPept) *Mycobacterium tuberculosis*  55%

[AAC46128.1|](http://www.ncbi.nlm.nih.gov/entrez/query.fcgi?cmd=Retrieve&db=Protein&list_uids=2130642&dopt=GenPept) *Bordetella bronchiseptica* 52%

[XP_658035.1|](http://www.ncbi.nlm.nih.gov/entrez/query.fcgi?cmd=Retrieve&db=Protein&list_uids=67516299&dopt=GenPept) *Aspergillus nidulans* 54%
