# Supplementary material for: Brucella suis urease encoded by ure1 but not ure2 is necessary for intestinal infection of BALB/c mice
Source: BMC Microbiol. 2007 Jun 19;7:57. doi: 10.1186/1471-2180-7-57 (PMC1983905; doi:10.1186/1471-2180-7-57)
Supplement: Additional File 2 — Sequences of primers (5' to 3') used to amplify ure genes. [file 1471-2180-7-57-S2.doc]

**Supplementary Material**

**Table 2. Sequences of primers (5’ to 3’) used to amplify ure genes**

**Primer name Sequence (5’ to 3’)**

UreaseONE-Forward CGACGCCGTAGGTAAATC

UreaseONE-Reverse TGAAATGGACATGGGTATCG

**UreaseTWO-Forward** GCTTGCCCTTGAATTCCTTTGTGG

**UreaseTWO-Reverse** ATCTGCGAATTTGCCGGACTCTAT

Ure-2-AB-Forward CGGGGATCCCATCACAATCGGCAAACA

Ure-2-AB-reverse CGGTCTAGAATGGCGCGAAGGAAGGTT 3'
